# Impact of Multi-Component Surrogates on the Performances, Pollutants, and Exergy of IC Engines

**DOI:** 10.3390/e24050671

**Published:** 2022-05-10

**Authors:** Kambale Mondo, Senda Agrebi, Fathi Hamdi, Fatma Lakhal, Amsini Sadiki, Mouldi Chrigui

**Affiliations:** 1 Research Unit of Mechanical Modeling, Energy and Materials, National School of Engineers of Gabes, University of Gabes, UR17ES47, Gabes 6029, Tunisia; augustin.kambalemondo@enig.u-gabes.tn (K.M.); fathi.hamdi@enig.u-gabes.tn (F.H.); lakhal.fatma@gmail.com (F.L.); mouldi.chrigui@enig.rnu.tn (M.C.); 2 Laboratoire de Modélisation Mécanique, Energétique et Matériaux, Institut Supérieur des Techniques Appliquées, 31 NDOLO, Kinshasa P.O. Box 6534, Congo; sadiki@ekt.tu-darmstadt.de; 3 Institute for Energy and Power Plant Technology, Technische Universität Darmstadt, 64287 Darmstadt, Germany

**Keywords:** diesel surrogate, reaction mechanism, RNG, four-stroke IC engine, exergy efficiency

## Abstract

Even though there is a pressing interest in clean energy sources, compression ignition (CI) engines, also called diesel engines, will remain of great importance for transportation sectors as well as for power generation in stationary applications in the foreseeable future. In order to promote applications dealing with complex diesel alternative fuels by facilitating their integration in numerical simulation, this paper targets three objectives. First, generate novel diesel fuel surrogates with more than one component. Here, five surrogates are generated using an advanced chemistry solver and are compared against three mechanisms from the literature. Second, validate the suggested reaction mechanisms (RMs) with experimental data. For this purpose, an engine configuration, which features a reacting spray flow evolving in a direct-injection (DI), single-cylinder, and four-stroke motor, is used. The RNG k-Epsilon coupled to power-law combustion models is applied to describe the complex in-cylinder turbulent reacting flow, while the hybrid Eulerian-Lagrangian Kelvin Helmholtz-Rayleigh Taylor (KH-RT) spray model is employed to capture the spray breakup. Third, highlight the impact of these surrogate fuels on the combustion properties along with the exergy of the engine. The results include distribution of temperature, pressure, heat release rate (HRR), vapor penetration length, and exergy efficiency. The effect of the surrogates on pollutant formation ($NO_{X}$, CO, $CO_{2}$) is also highlighted. The fifth surrogate showed 47% exergy efficiency. The fourth surrogate agreed well with the maximum experimental pressure, which equaled 85 Mpa. The first, second, and third surrogates registered 400, 316, and 276 g/kg fuel, respectively, of the total CO mass fraction at the outlet. These quantities were relatively higher compared to the fourth and fifth RMs.

## 1. Introduction

Compression ignition (CI) engines, also called diesel engines, are the most popular drive technology for the transportation sector as well as for power generation in stationary applications. The growing environmental regulations associated with fossil fuel combustion and the decreasing fossil fuel reserves, as well as the increasing demands for clean energy, have resulted in a progressing interest in the development of alternative renewable energy sources and even in the promotion of more sustainable, possibly carbon-free fuels. This implies that diesel family fuel combustion will remain of great importance in the foreseeable future, and it needs further research interest, in particular, in terms of further improving engine performances.

Focusing on the combustion process [1], it is important to master not only the complex in-cylinder flow behavior but also the effects of fuel composition on combustion properties and subsequent emissions. Complementary to experimental investigations, computational simulation is acknowledged to be essential for the development and implementation of novel technologies. This computer-based modeling, analysis, simulation, and optimization enables a cost-effective, efficient, and complementary worldwide approach to studying engineering applications and engineering new technical solutions when experimental measurement techniques are not available or investigations are too costly.

However, it is well-known that diesel and even gasoline fuels contain hundreds of chemical species, which makes it difficult to account for a detailed reaction mechanism (RM) that includes chemical species together with the complex fuel composition. This is also the case for diesel-alternative fuels. Therefore, surrogate fuels, which are simpler representations of these real fuels, gain relevance as they provide a better understanding of physico-chemical properties, including fundamental fuel composition and emission formation [1]. In this respect, they help researchers to characterize diesel fuels in many applications such as spray atomization, chemical kinetic modeling, and combustion simulation [1,2,3,4,5]. It turns out that for the analysis of a unique-component surrogate, e.g., n-dodecane and n-heptane, the physico-chemical properties are well understood. Even if such a single-component mechanism leads to the development of fundamental appreciative knowledge of diesel combustion, a single component fuel surrogate cannot fully represent the physico-chemical properties of a real diesel [5,6,7].

Nevertheless, previous studies have focused on reaction mechanisms with one component. They led to interesting results [8,9,10,11]. Banarge et al. [9] studied a reduced reactions kinetics mechanism (41 species and 132 reactions) with a single component n-heptane diesel surrogate. Analyses were carried out to establish the relationships between local temperature, equivalence ratio, and fuel–air mixing. Yoshikawa et al. [8] investigated, numerically, the diesel combustion regimes. They worked out an improved n-heptane detailed chemistry mechanism for modeling high- and low-temperature diesel fuel. It was found that the improved n-heptane reaction mechanism enhances the prediction of ignition delay for all cases, reproducing the cool flame heat release adequately [12].

Many former researchers have put their attention on the production of diesel surrogates, gasoline, and jet fuels. The reaction mechanisms were used in the validation of the fuel characteristic parameters [13,14]. Here, the main fuel parameters include density, evaporation characteristics (boiling range fuel energy content, flash point, vapor pressure), thermal conductivity, surface tension, and viscosity [15,16,17].

Mainstream transport fuels, such as diesel, gasoline, or jet A, are distillates of fossil crude oil. These multi-component fuels are composed of various mixtures of types of hydrocarbons (e.g., aromatics, alkenes, cyclic alkanes). In fact, physicochemical properties of each compound (e.g., boiling point, reaction rate, thermodynamics) should be accounted for to correctly model diesel fuel [18,19].

For that purpose, many researchers have pioneered an advanced group technique for diesel modeling. They have developed surrogate fuels and validated them for CFD application. The focus is on the production of kinetics reaction mechanisms using combustion data (e.g., CN, H/C molar ratio, ignition delay). Recently, Charles J. Mueller et al. [16] applied a new method to formulate surrogate fuels with representative characteristics of diesel. Much progress was made in the field of production of surrogate diesel and biodiesel. However, very scarce research has been conducted on the characterization of diesel in combustion. Yan et al. [20] examined the exergy losses in an n-heptane surrogate in an adiabatic reactor. Pathways to reduce the amount of exergy losses were conducted by evaluating the influences of oxygen concentration, equivalence ratio, initial temperature, and pressure. Zhang et al. [21] classified the chemical reactions in the fuel reaction mechanisms into four main stages and evaluated the chemical effects, dilution, and carbon dioxide addition on exergy losses for the four reaction stages. The same groups studied the exergy losses at the start of the ignition of alcohols and dimethyl ether (DME) blends. By analyzing the interaction effects between the reaction kinetics of alcohols and DME, it was found that the addition of methanol was more effective than ethanol in minimizing the exergy losses. Additionally, the combination of chemistry, kinetics, and the second thermodynamics law identified the contributions of reactions to the exergy losses. Li, Y. et al. [22,23] found that a detailed reaction mechanism could not be applied to a three-dimensional simulation of an IC engine due to the limitation of calculation capability. It is, therefore, necessary to develop fuel kinetic mechanisms with reduced sizes for engine combustion simulation. The complexity of RMs associated with 2-, 3-, and 4-component fuel leads to a stiff and complex chemistry, with thousands of species. For that purpose, multi-component surrogates that are produced from a detailed kinetic mechanism, Diesel_PAH_NOx_MFL2019 [24], were developed and investigated. The surrogates were applied in a 3D combustion chamber. Five surrogates were produced. Improvements and the validation of Chemkin models for the surrogate’s components were conducted with experimental results and carried out on a heavy-duty direct injection diesel engine. An energy–exergy analysis report was also produced.

In order to use complex diesel fuels, by facilitating their easy integration in numerical simulation, it appears essential to prepare a reliable strategy for deriving surrogate fuels with various components. Therefore, the present paper targets three objectives. The primary goal is to generate diesel fuel surrogates with more than one component. This is performed using an advanced chemistry solver, the Ansys-reaction workbench. Five surrogates with more than one component are generated and are compared against three mechanisms from the literature. Second, it aims at validating the suggested reaction mechanisms (RMs) with experimental data. For this purpose, an engine configuration is used, which features a reacting spray flow evolving in a direct-injection (DI), single-cylinder, and four-stroke motor, the so-called Sandia Lab engine configuration. The RNG k-Epsilon coupled to appropriate combustion models is applied to describe the complex in-cylinder turbulent reacting flow, while the hybrid Eulerian-Lagrangian Kelvin Helmholtz-Rayleigh Taylor (KH-RT) spray model is employed to capture the spray breakup. Third, it targets to highlight the impact of these surrogate fuels on the combustion properties, along with the exergy of the engine. The results include distribution of temperature, pressure, heat release rate (HRR), vapor penetration length, and exergy efficiency. The effect of the surrogates on pollutants formation ($NO_{X}$, CO, $CO_{2}$) is also highlighted together with the exergy efficiency.

The paper is organized as follows: First, an introduction and state of the art, including previous work in the literature, is presented. Then, the governing equations to model the combustion and flame speed as well as the production of fuel surrogates are introduced. In the third section, a presentation of the geometry and boundary conditions of the used configuration is highlighted. The fourth chapter deals with a detailed discussion of the results as well as the models’ validation. Finally, a general conclusion summarizes the work.

## 2. Governing Equations and Models

### 2.1. Transport Equations

Dealing with reacting spray, as featured in a (DI) diesel engine, a Eulerian-Lagrangian description approach is adopted in this paper. The RNG k-Epsilon model, as adapted in [25], is chosen for its effectiveness in strongly sheared flows to represent the turbulent carrier gas phase at lower computational costs. The following Favre-averaged transport equations to describe the carrier phase are given as

(1) $$\frac{\partial \bar{\rho }}{\partial t}+\nabla \cdot \left(\bar{\rho }\tilde{u}\right)={\dot{\bar{\rho }}}^{s}$$

(2) $$\frac{\partial \bar{\rho }\tilde{u}}{\partial t}+\nabla \cdot \left(\bar{\rho }\tilde{u}\tilde{u}\right)=-\nabla \bar{p}+\nabla \cdot \bar{\sigma }-\nabla \cdot \Gamma +{\bar{F}}^{S}+\bar{\rho }\bar{g}$$

(3) $$\frac{\partial \bar{\rho }\tilde{I}}{\partial t}+\nabla \cdot \left(\bar{\rho }\tilde{u}\tilde{I}\right)=-\bar{p}\nabla \cdot \tilde{u}-\nabla \cdot \bar{J}-\nabla \cdot H+\bar{\rho }\tilde{\varepsilon }+{\dot{\bar{Q}}}^{C}+{\dot{\bar{Q}}}^{S}$$

where Equations (1)–(3) are equations for the density, momentum, and energy, respectively. The notations (bar) stand for the Reynolds-and (tilde) for Favre-averaging.

In particular, ${\dot{\bar{\rho }}}^{s}$ is the averaged source term related to spray evaporation, $\tilde{u}$ is the Favre averaged velocity, while ρ represents the density. The quantity $\bar{\sigma }$ represents the averaged viscous shear stress,$\;{\bar{F}}^{s}$ is the source term due to spray injection, and Γ is the stress tensor that accounts for the effects of ensemble averaging. The quantity $\bar{J}=-\lambda \nabla \bar{T}-\bar{\rho }D\underset{k}{\sum }{\tilde{h}}_{k}\nabla {\bar{y}}_{k}$ is the heat flux vector, with λ as the thermal conductivity, *T* as the fluid temperature, and ${\tilde{h}}_{k}$ as the specific enthalpy of species. The quantities ${\dot{\bar{Q}}}^{C}$,${\dot{\bar{\;Q}}}^{S}$ are source terms due to the reaction heat release rate and spray interaction, respectively. Note that H is the convection filtered term. The term $\bar{\rho }\tilde{I^{\prime \prime }u^{\prime \prime }}$ represents the turbulent transport of the scalar quantities in the energy equation.

In addition to the mass, momentum, energy, and species transport equations, the following two equations for k and Epsilon are solved to close the momentum equation within the RNG k-Epsilon framework (see details in [26,27]):

(4) $$\frac{\partial \bar{\rho }\tilde{k}}{\partial t}+\nabla \cdot \left(\bar{\rho }\tilde{u}\tilde{k}\right)=-\frac{2}{3}\bar{\rho }\tilde{k}\nabla \cdot \tilde{u}+\left(\bar{\sigma }-\Gamma \right):\nabla \tilde{u}+\nabla \cdot \left[\frac{\left(\mu \right)+\mu_{T}}{Pr_{k}}\nabla \tilde{k}\right]-\bar{\rho }\tilde{\varepsilon }+{\dot{\tilde{W}}}^{S}$$

(5) $$\frac{\partial \bar{\rho }\tilde{\varepsilon }}{\partial t}+\nabla \cdot \left(\bar{\rho }\right)\tilde{u}\tilde{\varepsilon }=-\left(\frac{2}{3}C_{\varepsilon 1}-C_{\varepsilon 3}\right)\bar{\rho }\tilde{\varepsilon }\nabla \cdot \tilde{u}+\nabla \cdot \left[\frac{\left(v+v_{T}\right)}{Pr_{\varepsilon }}\nabla \tilde{\varepsilon }\right]+\frac{\tilde{\varepsilon }}{\tilde{k}}\left[C_{\varepsilon 1}\left(\bar{\sigma }-\Gamma \right):\nabla \tilde{u}-C_{\varepsilon 2}\bar{\rho }\tilde{\varepsilon }+C_{S}{\dot{\tilde{W}}}^{S\;}\right]-\bar{\rho }R,$$

where *R* is defined as:

(6) $$R=\frac{C_{\mu }\eta^{3}\left[1-\eta /\eta_{O}\right]}{1+\beta \eta^{3}}\frac{{\tilde{\varepsilon }}^{2}}{\tilde{k}}$$

$\eta =S\frac{k}{\tilde{\varepsilon }}$, $S={\left(2\bar{S}:\bar{S}\right)}^{1/2}$ and $\bar{S}$ is the mean strain rate tensor $\bar{\;S}=\frac{1}{2}\left(\nabla \tilde{u}+{\left(\nabla \tilde{u}\right)}^{T}\right)$.

The values of the model $Pr_{k}$, $Pr_{\varepsilon }$, $C_{\varepsilon 1}$ and $C_{\varepsilon 2}$ used in the RNG version equal 0.72, 0.72, 1.42, 1.68, respectively [25]. In particular, $C_{\varepsilon 3}$ is calculated following the Equation (7).

(7) $$C_{\varepsilon 3}=\frac{-1+2C_{\varepsilon 2}-3m\left(n-1\right)+{\left(-1\right)}^{\delta }\sqrt{6}C_{\mu }C_{\eta }\eta }{3}$$

*m* = 0.5, *n* = 1.4 for an ideal gas, and $\;C_{\eta }=\frac{\eta \left(1-\eta /\eta_{o}\right)}{1+\beta \eta^{3}}$,

with$\;\delta =\{\begin{array}{c} 1,\;if\;\nabla \tilde{u}<0;\\ 0,if\;\nabla \tilde{u}>0. \end{array}$

Here, the constants $C_{\mu }$ = 0.0845, $\eta_{o}$ = 4.38 and β  = 0.012 [27]. It is worth mentioning that ${\dot{\tilde{W}}}^{S}$ accounts for the contribution due to evaporating droplets.

For the dispersed droplet phase, the Lagrangian approach is used. The droplet motion equation and the droplet temperature, including the evaporation Discrete Multi-Component Model (DMC), following [28], are solved. The Kelvin Helmholtz-Rayleigh Taylor (KH-RT) spray model is employed to capture the spray breakup [12].

To avoid repetition without preventing the self-consistency of the paper, these models are not reproduced here as they are comprehensively documented in the literature, especially in [2,29,30].

### 2.2. Combustion Models

The gas phase, in the combustion chamber, is modeled as individual gas components or species. Note that this composition is changed during the combustion in CI Engine due to molecular diffusion, flow convection, turbulent transport, and turbulence chemistry interaction.

(8) $$\frac{\partial {\bar{\rho }}_{m}}{\partial t}+\nabla \cdot \left({\bar{\rho }}_{m}\tilde{u}\right)=\nabla \cdot \left[\bar{\rho }D\nabla {\bar{y}}_{m}u\right]+\nabla \left({\bar{\rho }}_{m}\tilde{u}-\bar{\rho_{m}u}\right)+{\dot{\bar{\rho }}}_{m\;}^{C}+{\dot{\bar{\rho }}}_{m\;}^{s}$$

where ρ, and $\vec{u}$ are the density and the flow velocity vector, respectively. The subscript m is the species index, D denotes the mixture-average molecules diffusions, $y_{m}=\rho_{m}/\rho$ is the mass fraction of species m, and ${\dot{\bar{\rho }}}_{m\;}^{s}$ is the sprays’ vaporization. The quantity ${\dot{\bar{\rho }}}_{m\;}^{C}$ is the source term due to chemical reactions, which is calculated using the following equation:

(9) $${\dot{\bar{\rho }}}_{m\;}^{C}=W_{m}\overset{I}{\underset{i=1}{\sum }}{\dot{\omega }}_{mi},$$

where, ${\dot{\omega }}_{mi}$ represents the production rate of the mth species in the ith reaction; it is modeled as presented [24]:

(10) $${\dot{\omega }}_{mi}=\left({v^{\prime \prime }}_{mi}-{v^{\prime }}_{mi}\right)q_{i}$$

The term $q_{i}$ represents the rate of progress of specified reaction *i*, while $v^{\prime \prime }and\;v^{\prime }$ are the stoichiometric coefficients for forward and backward reactions.

In this k-Epsilon framework, a gradient assumption is used [31] for the turbulent transport of the scalar quantities:

(11) $$\bar{\rho u^{\prime \prime }\phi^{\prime \prime }}=-\Gamma_{\phi ,T}grad{\tilde{\phi }}_{g},$$

where $\Gamma_{\phi ,T}$ is the turbulent exchange coefficient of ϕ. For mass transfer, $\Gamma_{Y_{m},T}=\bar{\rho }D_{T}$, with the turbulent diffusion coefficient $D_{T}\;$given by $D_{T}=\mu_{T}/\left({\bar{\rho }\mathrm{Sc}}_{T}\right)$, where ${\mathrm{Sc}}_{T}$ is the turbulent Schmidt number. For energy transfer, the turbulent exchange coefficient $\Gamma_{\phi ,T}={\;K}_{T}/c_{p}$, with the turbulent thermal conductivity $K_{T}$ given by $K_{T}={\;\mu }_{T}c_{p}/{\mathrm{Pr}}_{T}$, where ${\;\mathrm{Pr}}_{T}$ is the turbulent Prandtl number.

#### Turbulent Flame Speeds

A Favre-averaged G-equation is used to track the flame front as well as the flame position. This model was proposed by Peters [32] for a premixed flame. For the application of the G-equations on an IC engine (non-premixed flame), Peters’s model was extended by Tan [33] and by Liang et al. [34] to account for diffusion flame properties. During the combustion, the G-equation, G(x, t), equals zero at the flame front in the unburnt region G(x, t) < 0, while G(x, t) > 0 means that the region is burnt [12]. The set of the equations are written as:

(12) $$\frac{\partial \tilde{G}}{\partial t}+\left(\tilde{\vec{u}}-{\vec{u}}_{vertex}\right)\nabla \tilde{G}=\frac{{\bar{\rho }}_{u}}{{\bar{\rho }}_{b}}S_{T}^{O}\left|\nabla \tilde{G}\right|-D_{T}\tilde{\kappa }\left|\nabla \tilde{G}\right|$$

(13) $$\frac{\partial \tilde{G^{\prime \prime 2}}}{\partial t}+\tilde{\vec{u}}\nabla \tilde{G^{\prime \prime 2}}=\nabla_{∥}\cdot \left(\frac{{\bar{\rho }}_{u}}{{\bar{\rho }}_{b}}D_{T}\nabla_{∥}\tilde{G^{\prime \prime 2}}\right)+2D_{T}{\left(\nabla \tilde{G}\right)}^{2}-C_{S}\frac{\tilde{\varepsilon }}{\tilde{k}}\tilde{G^{\prime \prime 2}}$$

In these equations, $\nabla_{∥}$ denotes the tangential gradient operator,$\;\vec{u}$ is the fluid velocity, ${\vec{u}}_{vertex}$ is the velocity of the moving vertex, while $\rho_{u}$ and $\rho_{b}$ represent the average densities of the unburned and burned mixtures, respectively. The term $D_{T}$ is the turbulent diffusivity, $\tilde{\kappa }$ is the Favre mean flame front curvature, and $C_{S}$, $a_{4}$, $b_{1}$, and $b_{3}$ are model constants provided in [8,12,35,36]. The quantities $\tilde{k\;}$ and $\tilde{\varepsilon \;}$ are the Favre mean turbulent kinetic energy and its dissipation rate from the RNG k−ε model, while $u^{\prime \;}$is the turbulence velocity fluctuation.

The turbulent flame $S_{T}^{O}$, used in the G-equation, is obtained through the following equation [8,12,35,36]:

(14) $$\frac{S_{T}^{o}}{S_{L}^{o}}=1+I_{P}\left\{-\frac{a_{4}b_{3}^{2}}{2b_{1}}\frac{l_{I}}{l_{F}}+{\left[{\left(\frac{a_{4}b_{3}^{2}}{2b_{1}}\frac{l_{I}}{l_{F}}\right)}^{2}+a_{4}b_{3}^{2}\frac{u^{\prime }}{S_{L}^{o}}\frac{l_{I}}{l_{F}}\right]}^{1/2}\right\}$$

The quantities $l_{I}\;$and $l_{F}\;$are the turbulence integral length scale and the laminar flame thickness, respectively. The term $S_{L}^{o}\;$denotes the laminar flame speed, which is calculated as [37]:

(15) $$S_{L}^{O}=S_{L,ref}^{O}{\left(\frac{T_{u}}{T_{u,ref}}\right)}^{\alpha }{\left(\frac{p}{p_{ref}}\right)}^{\beta }F_{dil}$$

The subscript *ref* means the reference condition (typically at 298 K and 1 atm.), and the superscript 0 represents the planar and the unstretched flame. $F_{dil}$ is a factor accounting for the diluent’s effect.

### 2.3. Surrogate Fuel Generation

In all these models, it is essential to have the reaction mechanisms of the fuel used. For this purpose, five surrogate fuels are generated. They are: (1) Predicted-1 (30% of aromatics and 70% n-alkanes) with 1883 species and 8254 reactions; (2) Predicted-2 (aromatics, cycloalkanes, iso-alkanes, and n-alkanes) with 1883 species and 8254 reactions; (3) Predicted-3 (aromatics, iso-alkanes, and n-alkanes) with 1883 species and 8254 reactions; (4) Predicted-4 (aromatics, 1 ether, 1 iso-alkane, and 2 composition n-alkanes) with 445 species and 3562 reactions; (5) Predicted-5 (1 component of aromatics, 1 cycloalkane, and 2 composition n-alkanes) with 5384 species and 68,885 reactions. The percentage of each component is mentioned in the following sections.

In addition, three mechanisms from the literature, which are n-dodecane with 65 species and 363 reactions [11,38], n-heptane with 159 species and 770 reactions [3,39], and a diesel fuel surrogate with 163 species and 887 reactions [1], are also studied.

First, the surrogate blend optimizer will be introduced. Then, the surrogate reaction components and blend formulation will be presented.

#### 2.3.1. Surrogate Blend Optimization Formulation

**Figure 1:**
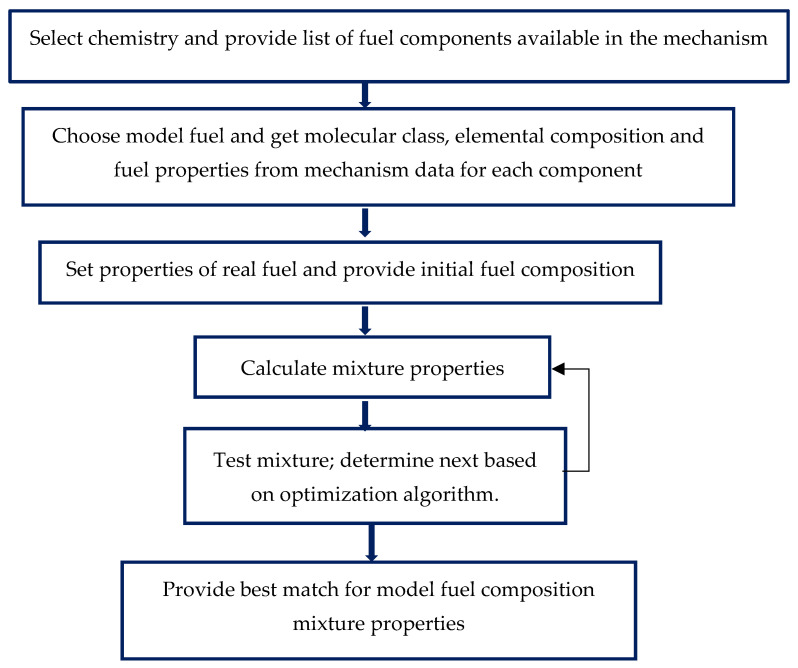
Workflow for the Surrogate Blend Optimizer.

The Surrogate Blend Optimizer (SBO) optimizes the composition of a fuel model. It is performed to match the specified properties of an actual fuel. The production of surrogate fuel is done using several steps, as shown in the algorithm (Figure 1). First, select the base chemical reaction mechanism, which is Diesel_PAH_NOx_MFL2019. This is a detailed reaction mechanism developed and validated by Ansys [40]. It involves 5384 species and 68,885 reactions. Second, set the composition of the reference fuel model. Third, introduce the properties of the actual fuel to be matched with an acceptable tolerance. Fourth, calculate the properties of the mixture and obtain the reaction mechanism for which the properties are then compared to the actual fuel properties. Finally, the fuel surrogate can be used as a detailed reaction mechanism for numerical simulation or/and could be reduced further.

#### 2.3.2. Surrogate Diesel Fuel

The workflow is as follows: First, the creation of a diesel baseline surrogate is required. The fuel reaction mechanism properties for various surrogates are given in Table 1 [2]. The value of the cetane number (CN) is around 50 for all surrogates. The Threshold Soot Index (TSI) is set to 31. The used values for the baseline diesel fuel, which are considered target values, are LHV, density, viscosity, molar H/C, and T10 to T90. Here, a weighting factor of 1.0 is used. It is found that the Diesel Fuel Surrogate 1 (Predicted-1) is able to match only the LHV and H/C compared with measured diesel fuel. The Diesel Fuel Surrogate 2 and 4 (Predicted-2 and -4) are able to match four properties of the target baseline diesel fuel.

### 2.4. Energy and Exergy Analysis

#### 2.4.1. Energy Analysis

The energy balance relationship is based on the quantitative analysis method [41,42,43]. For a diesel engine with an operating cycle in the full equilibrium state, the energy equation is given by:

(16) $$\frac{dQ_{F}}{dt}=\frac{dQ_{E}}{dt}+\frac{dQ_{C}}{dt}+\frac{dW}{dt}+\frac{dQ_{M}}{dt}$$

where $Q_{F}$ is the heat release energy of the combustion of diesel fuel (J), W is the output energy (J), $Q_{M}$ is the miscellaneous or loss energy (J), and t is the time (s). The heat release rate in diesel fuel combustion is:

(17) $$\frac{dQ_{F}}{dt}=LHV{\dot{m}}_{f}$$

where LHV is the low heat value (${J\;\mathrm{kg}}^{-1}$), and$\;{\dot{m}}_{f}$ is mass flow rate of diesel fuel $\left({\mathrm{kg}\;s}^{-1}\right)$.

The convection and radiation energy from the engine surface are classified in$\;Q_{M}$ [44]. The exhaust energy is:

(18) $$\frac{dQ_{E}}{dt}={\dot{m}}_{Exhaust}\left(C_{pE}T_{E}-C_{pI}T_{I}\right)$$

Both $C_{pE}$ and $T_{E}$ are constant pressure specific heat capacity $\left({J\;\mathrm{kg}}^{-1}{\;K}^{-1}\right)\;$and temperature of exhaust gas (K), respectively. $C_{pI}$ is the constant pressure specific heat capacity $\left({J\;\mathrm{kg}}^{-1}{\;K}^{-1}\right)$.$\;T_{I}$ is the temperature of the intake air (K). $C_{p}$ is the constant pressure specific heat capacity of the mixture [45,46]. For every simple substance, $C_{p}$ of the mixture is related to temperature and it is obtained by thermodynamical interpolation as:

(19) $$C_{p}=\overset{k}{\underset{i=1}{\sum }}C_{pi}X_{i}$$

(20) $$X_{i}=\frac{m_{i}}{m_{exhaust}}$$

The $m_{exhaust}$, k, $C_{pi}$*,* $m_{i},$ and $X_{i}\;$are the exhaust mass, the number of components in the exhaust, constant pressure specific heat (J kg^−1^ K^−1^), mass (kg), and mass fraction of the ith single substance in the mixture, respectively.

The heat transfer energy to the coolant fluid from combustion gas is:

(21) $$\frac{dQ_{C}}{dt}={\dot{m}}_{c}C_{pc}\left(T_{Co}-T_{CI}\right),$$

where ${\dot{m}}_{c}$ is the mass flow rate of the coolant fluid of coolant $\left({\mathrm{kg}\;s}^{-1}\right)$,$\;C_{pc}$ is the specific heat capacity $\left({J\;\mathrm{kg}}^{-1}{\;K}^{-1}\right)$, $T_{Co}$ is the coolant fluid temperature outlet, and $T_{CI}$ is the coolant fluid temperature inlet.

#### 2.4.2. Exergy Analysis

Exergy analysis is the key concept of the viability of a diesel engine [6,45,47,48]. It gives the quantification of how fuel is transformed in the CI engine cycle [49]. Diesel engine qualitative balance for a whole steady-state of the cycle is given by the following equation:

(22) $$\frac{dE_{F}}{dt}=\frac{dE_{E}}{dt}+\frac{dE_{C}}{dt}+\frac{dE_{W}}{dt}+\frac{dE_{D}}{dt}$$

In this section $E_{F}$ is the fuel exergy (J), $E_{W}$ is the work (J), and $E_{D}$ is the exergy destruction (J).

The exergy of the fuel (CxHy) is as follows [50]:

(23) $$\frac{dE_{F}}{dt}=LHV\left(1.04224+0.011925\frac{y}{x}-\frac{0.042}{x}\right){\dot{m}}_{f},$$

where y and x are the numbers of hydrogen and carbon atoms contained in the fuel.

#### 2.4.3. Exergy Efficiency

**Figure 2:**
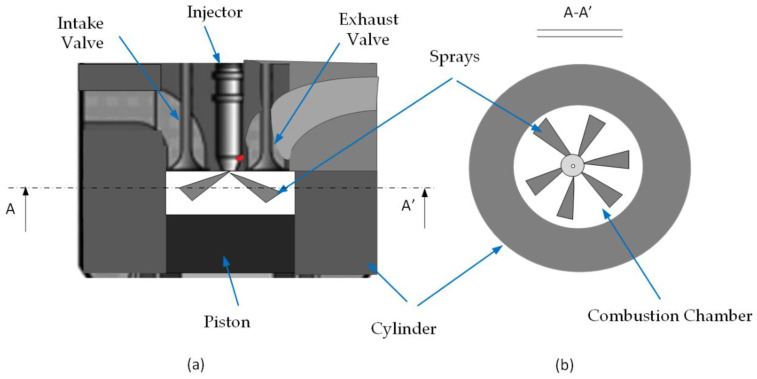
Principle of Engine Setup: (**a**) CI Engine Combustion Chamber. (**b**) Bottom View.

Exergy efficiency gives a better understanding of diesel engine performances [49,51]. Exergy efficiency is determined by the ratio of total power recovered by the diesel engine to the total chemical exergy rate input:

(24) $$\eta_{E}=\frac{{\dot{E}}_{W}}{{\dot{E}}_{F}}\;,$$

in which ${\dot{E}}_{W}$ is the rate of exergy associated with the work and ${\dot{E}}_{F}$ represents the exergy rate of fuel combustion. This modeling approach will be validated in a direct-injection (DI), single-cylinder, and four-stroke motor (Figure 2) [12].

## 3. Sandia Optical Engine Specification

The specifications of the engine are presented in Table 2. The compression ratio equals 16:1.

### 3.1. Computational Domain

**Figure 3:**
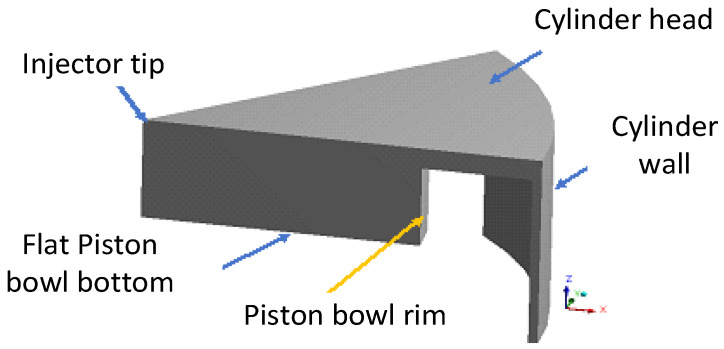
Geometry of the IC engine combustion chamber.

The schematics of the Cummins N-14 direct injection (DI) diesel engine’s geometry are illustrated in Figure 3. To reduce the computational costs, a periodic sector of the geometry is used. Since the combustion chamber’s geometry has 8 holes of injection, the computational domain is divided into 8 periodic sectors. Here, a 45-degree wedge sector of the whole geometry is simulated.

### 3.2. Boundary Conditions

**Figure 4:**
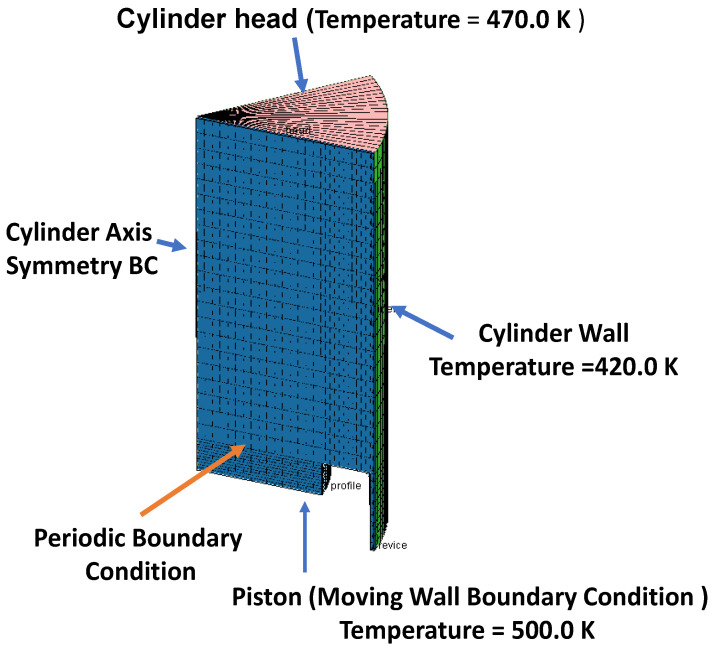
Boundary conditions used in the computational domain at −180 ATDC.

The computational grid is conducted using Ansys-forte. The geometry and computational mesh are shown in Figure 3 and Figure 4, respectively. The same operating conditions as the experimental work are used [8]. The setup values for the simulations are presented in Table 3.

The mesh structure of the computational domain is outlined in Figure 4. It contains 11,319 cells, enough to ensure grid-independent solutions. The computational domain is a 45-degree sector considering a 1-hole injector spray nozzle, as shown in Figure 4.

## 4. Results and Discussion

The present study is compared with the experimental results from the literature [8]. The comparison includes pressure, heat release, and vapor penetration. The in-cylinder pressure, temperature, and HRR at various degrees of the crankshaft are identified as a function of the crank angle.

### 4.1. Pressure

**Figure 5:**
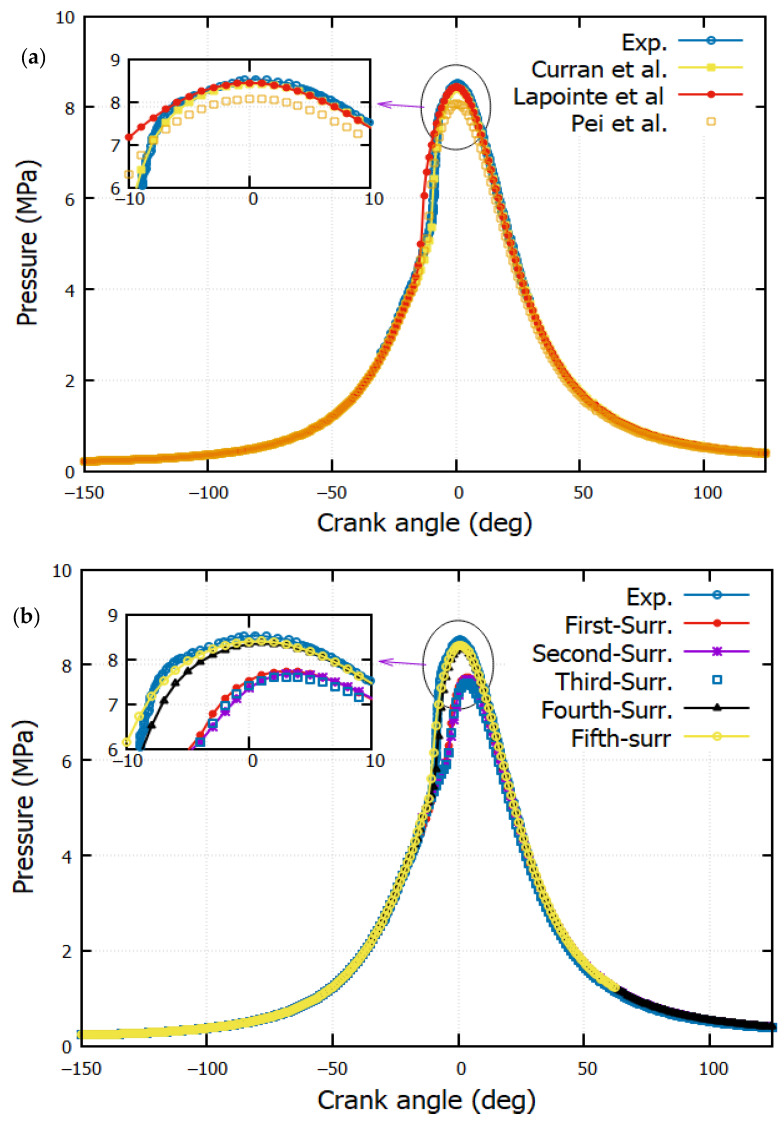
Comparison of in-cylinder experimental and simulation pressure in the CI engine: (**a**) Experiment against reactions mechanisms from literature (Curran et al. [39], Lapointe et al. [38] and Pei et al. [1]). (**b**) Experiment against predicted reactions mechanisms (The first-Surrogate, Second-Surrogate, Third-Surrogate, Fourth-Surrogate and Fifth-Surrogate).

**Figure 6:**
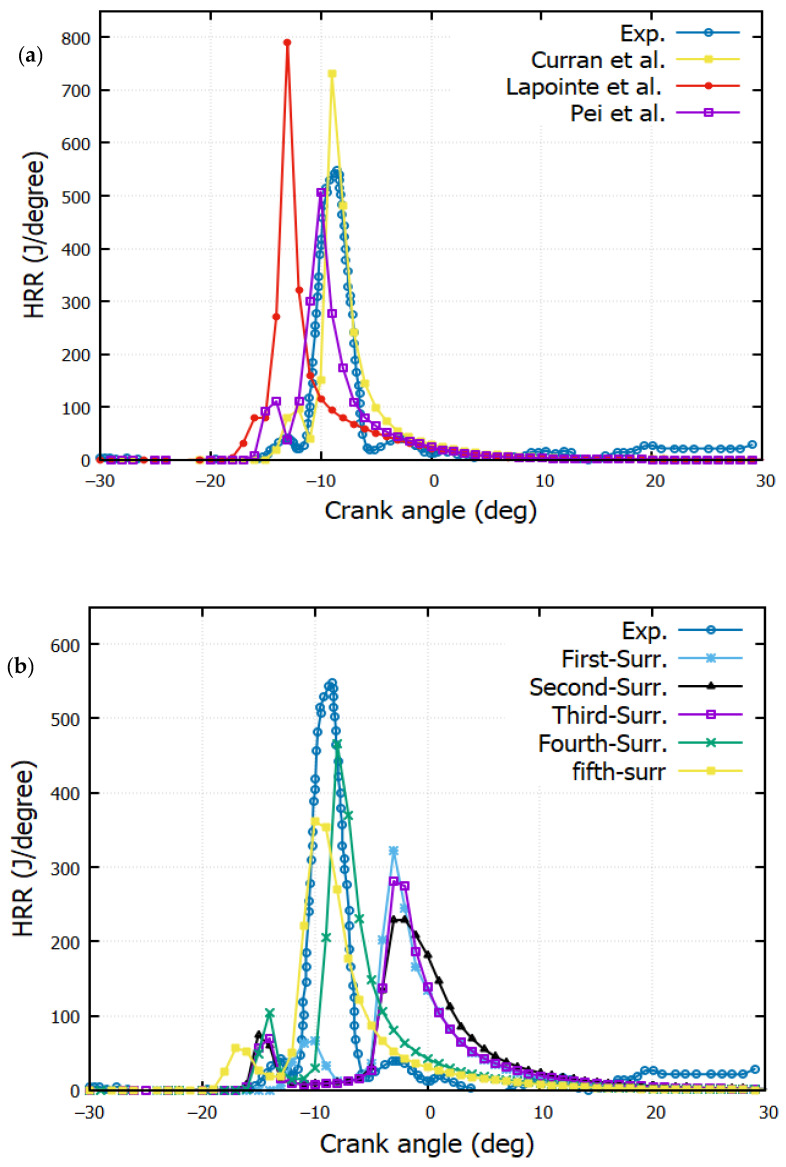
Variation of the rate of heat release against crank angle: (**a**) Experiment against reactions mechanisms from litterature (Curran et al. [39], Lapointe et al. [38] and Pei et al. [1]). (**b**) Experiment against predicted reactions mechanisms (The first-Surrogate, Second-Surrogate, Third-Surrogate, Fourth-Surrogate and Fifth-Surrogate).

Figure 5 shows the simulation results for the n-heptane mechanism in order to validate the numerical model. Figure 5a,b outline a comparison between the measured and the simulated pressure, in which a good agreement is observed. A gap between the experiment against the first, second, and third surrogates is noticed. This is caused by the delay of heat release, as shown in Figure 6. A remarkable difference in the prediction of ignition delay, which is the interval between the start of injection and the start of combustion, using the first, second, and third surrogates is noticeable. The start of combustion depends on the cetane number, which has a lower value for these surrogates.

### 4.2. Heat Release Rate (HRR)

The variation of the heat release rate (HRR), along the crank angle, is depicted in Figure 6. The variation of the heat release rate (HRR) along the crank angle is depicted in Figure 6. The HRR gives a hint about the rapidity of the combustion. The maximum heat release rates for diesel are 550, 800, 730, 510, 470, and 370 J/°CA for the experiment, n-dodecane (Lapointe et al. [38]), n-heptane (H.J. Curran [39]), the surrogate (Pei et al. [1]), Predicted-4, and Predicted-5, respectively.

Delays in the combustion, leading to a gap, are observed between the experiment and the first, second, and third surrogate reaction mechanisms. The auto-ignition of n-dodecane [38] takes place some milliseconds after the start of injection. The combustion is divided into two phases; first, a tiny increase of the HRR, caused by the interaction between fuel and air, is registered. Second, the diffusion turbulent combustion increases as long as the air–fuel mixture increases.

At the second stage, the HRR reaches a peak value. This is due to the increase in the air–fuel equivalence ratio. The remarkable difference between the different surrogates is owed to the different physicochemical properties of each component of the target fuel. The properties of each component have a remarkable influence on chemical heat release.

### 4.3. Vapor Penetration

**Figure 7:**
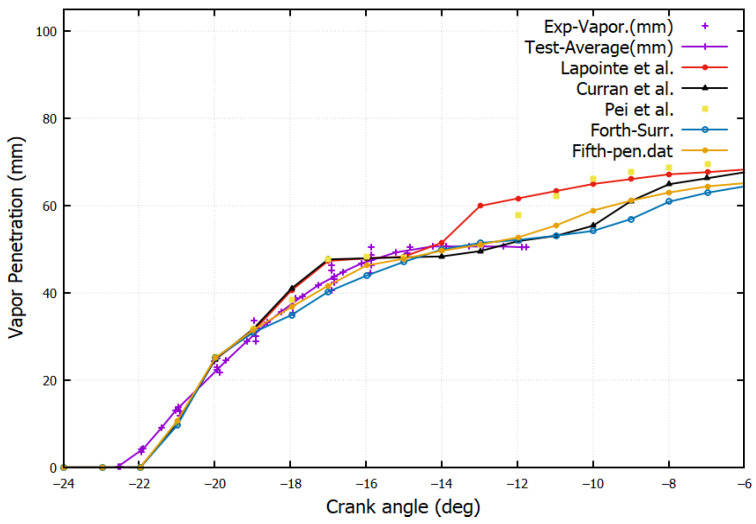
Vapor penetration (VP) for various fuel surrogates compared with experiments (Curran et al. [39], Lapointe et al. [38] and Pei et al. [1], Fourth-Surrogate and Fifth-Surrogate).

Figure 7 shows a comparison of vapor penetration between the experimental and the simulation results. Small discrepancies are observed, yet the profiles of vapor penetration for the different mechanisms change in good pace with the measurements. Starting from CA = −14°, the prediction of liquid penetration by the Pei et al. [1] and Lapointe et al. [38] surrogates are larger. This produces a higher rate of heat release and vaporization rate.

### 4.4. Maximum and Mean Temperatures

**Figure 8:**
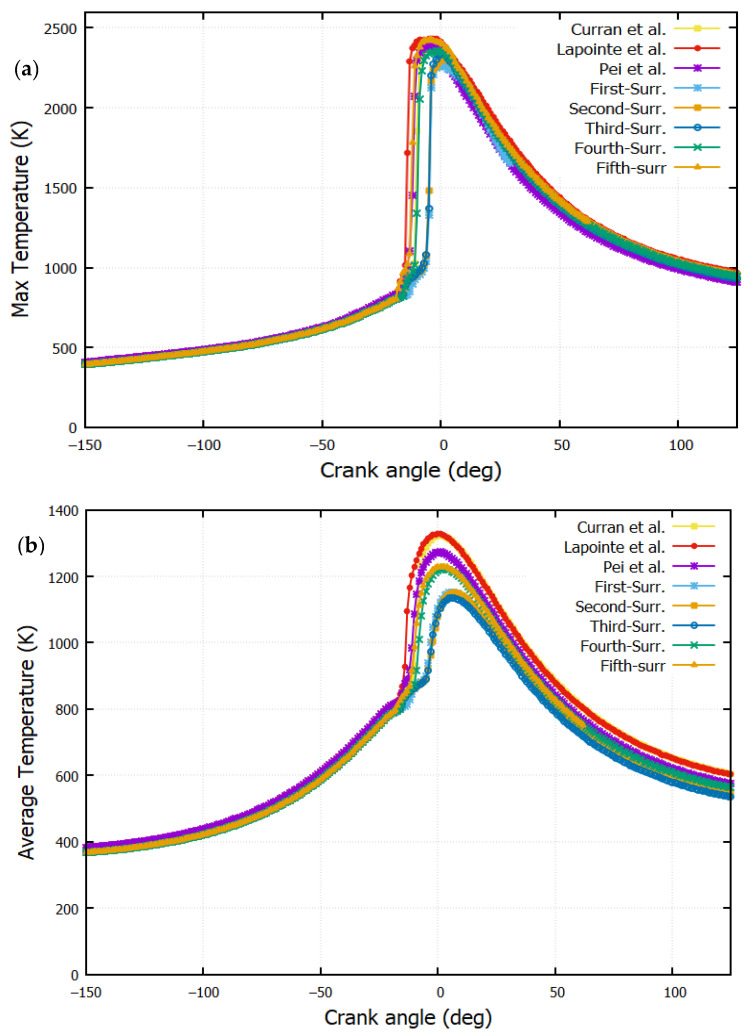
Variation of temperature with crank angle (**a**) maximum. (**b**) mean. (Curran et al. [39], Lapointe et al. [38] and Pei et al. [1], The first-Surrogate, Second-Surrogate, Third-Surrogate, Fourth-Surrogate and Fifth-Surrogate).

Figure 8a,b depict the maximum and the mean temperature, respectively. The evolution of the average temperature increases close to the TDC. The injection starts at −22° ATDC. Different combustion starts are observed for the different reaction mechanisms.

The highest maximum temperature is observed for n-dodecane (Lapointe et al. [38]); this is caused by its important cetane number (CN) and low auto-ignition temperature. This mechanism is followed by n-heptane (H.J. Curran [39]), which ignites rapidly due to its low combustion point. For each reaction mechanism, the maximum temperature value is reached at the same CA. It is when the piston approaches the top dead center (TDC). Differences are observed around the TDC. By the end of the piston motion (CA = 120°), the mean temperatures are almost identical.

### 4.5. Temperature Contours

**Figure 9:**
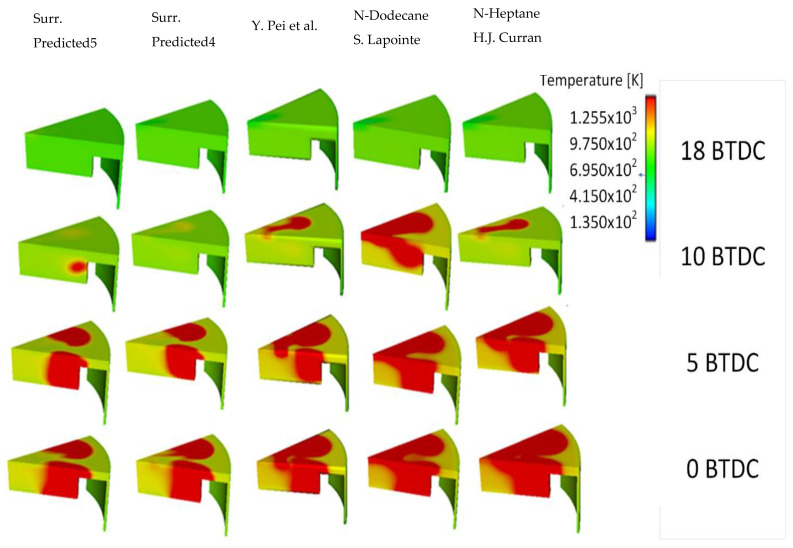
Temperature contour comparison of different surrogates and RMs at various crank angles of 18°, 10°, 5°, and 0° BTDC (SOI: −22 ATDC) (Curran et al. [39], Lapointe et al. [38] and Pei et al. [1], Fourth-Surrogate and Fifth-Surrogate).

Figure 9 shows a rapid development of the combustion for n-dodecane and n-heptane RMs. A delay is noticed for fuel surrogates at 5° BTDC. The rapid combustion of the air–fuel mixture causes a spike. A sudden rise is observed in the temperature and the heat release rate at 10° BTDC. The temperature remains almost constant during admission (temperature of the fresh gases), then it increases slowly during the compression phase.

A small variation is obtained at 18° BTDC; it is due to the elemental influence of the cold diesel fuel injection. A sudden jump is observed afterward, that is, due to the diffusion of the flame heat reaction. The large value of average temperature equals 1200 K, while the maximum temperature equals 2400 K. They are registered by n-heptane. Despite different values of HRR for the different mechanisms, it is observed that the qualitative distributions are not highly significant for temperature and pressure (Figure 5 and Figure 8a,b).

### 4.6. CO Mass Fraction

**Figure 10:**
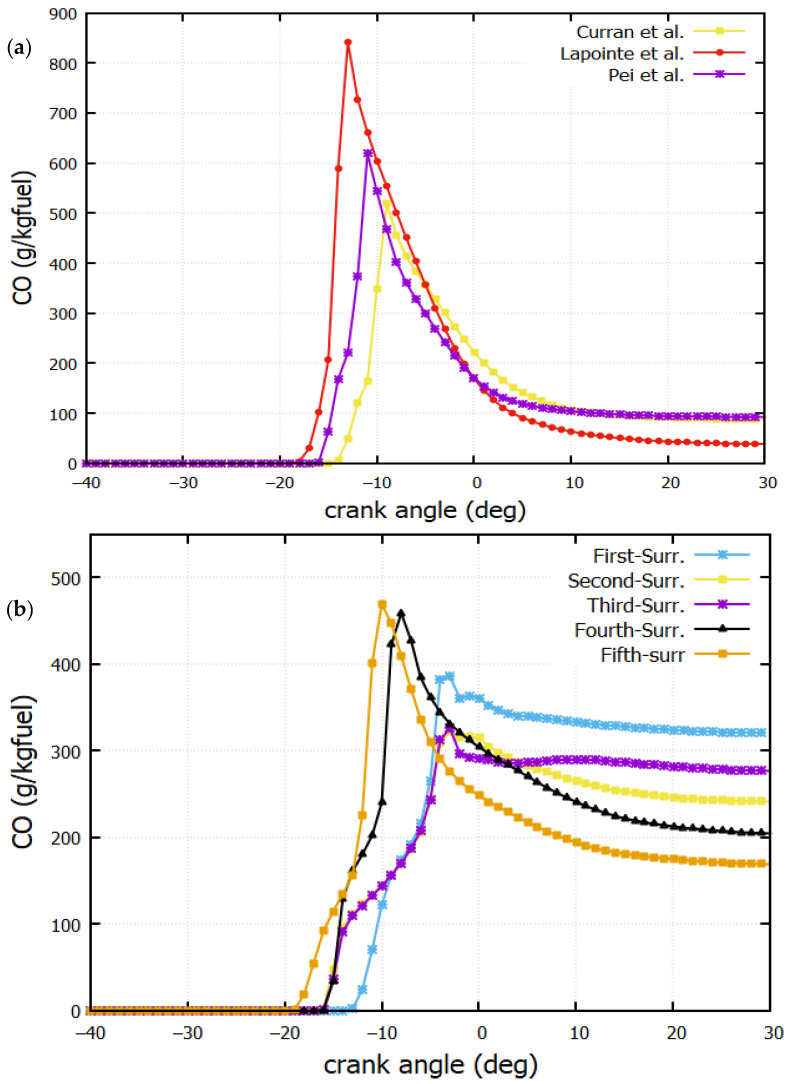
CO mass fraction against crank angle for (**a**) Reactions mechanisms from litterature (Curran et al. [39], Lapointe et al. [38] and Pei et al. [1]); (**b**) Predicted reactions mechanisms (The First-Surrogate, Second-Surrogate, Third-Surrogate, Fourth-Surrogate and Fifth-Surrogate).

Figure 10a,b show the variation of the CO mass fraction with respect to the CA. The CO emissions are formed either in insufficient $O_{2}\;$or in a locally rich (air/fuel) mixture. At the temperature peaks (Figure 8), the highest CO mass fraction is observed. The n-dodecane, S. Lapointe [38], shows the maximum peak at CA = 13° BTDC. For CA > 20° ATDC, the CO mass fraction remains constant for all mechanisms. Predicted-1 records the maximum CO=320 g/kg fuel mass fraction (Figure 10a).

### 4.7. Unburned Hydrocarbon (UBHC) Mass Fraction

**Figure 11:**
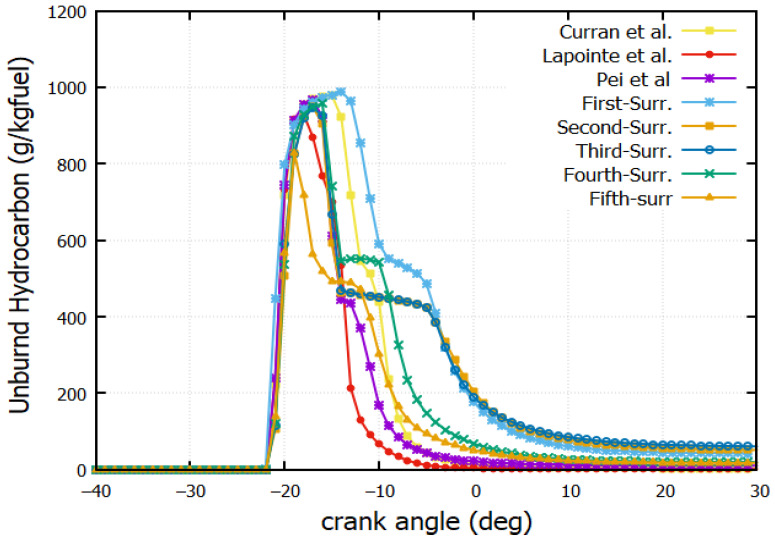
Unburned Hydrocarbon mass fraction against crank angle (Curran et al. [39], Lapointe et al. [38] and Pei et al. [1], The First-Surrogate, Second-Surrogate, Third-Surrogate, Fourth-Surrogate and Fifth-Surrogate).

Figure 11 shows the output of unburned hydrocarbon (UBHC) for all reaction mechanisms. As expected, the UBHC emissions decrease for higher values of temperature and heat release rate. The quantity of unburned hydrocarbons contained in the exhaust gas depends on the homogeneity of the air/fuel mixture, which plays an important role in the propagation of the flame. Unburned hydrocarbons originate from squish volumes and crevices. The flame is always extinguished in these regions as they are close to the wall. A delay of CA = 4° is noted in the UBHC production for the first surrogate compared to n-dodecane (Lapointe et al. [38]). This agrees with the HRR (Figure 6a,b).

### 4.8. Energy Efficiency

**Figure 12:**
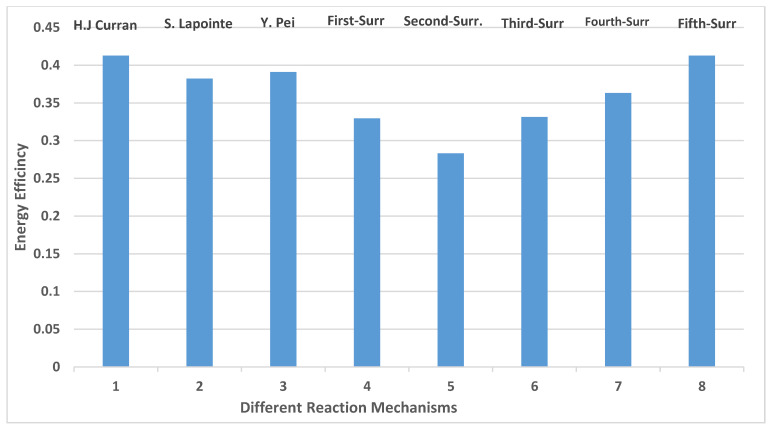
Energy efficiency for different reaction mechanisms (RMs) (Curran et al. [39], Lapointe et al. [38] and Pei et al. [1], The First-Surrogate, Second-Surrogate, Third-Surrogate, Fourth-Surrogate and Fifth-Surrogate).

Energy efficiency is important to quantify ICE performances. In Figure 12, higher energy efficiency is observed for the fifth reaction mechanism. It equals 41.3%. It is followed by n-dodecane (H.J. Curran) with 41%. The lower energy value is recorded by the second surrogate, which equals 28%.

### 4.9. Exergy Efficiency

**Figure 13:**
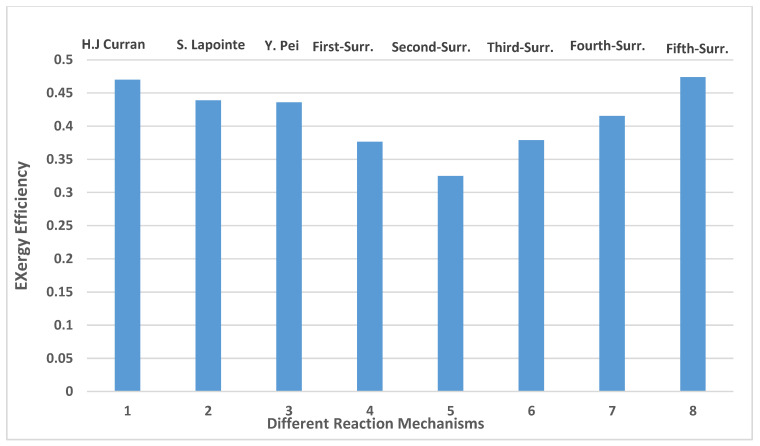
Exergy efficiencies of different reactions mechanisms (Curran et al. [39], Lapointe et al. [38] and Pei et al. [1], The First-Surrogate, Second-Surrogate, Third-Surrogate, Fourth-Surrogate and Fifth-Surrogate).

Exergy efficiency is a qualitative analysis, which allows a good understanding of the recovering energy by a diesel engine. The calculation method for exergy efficiency is conducted through Equation (23) using the values in Table 4. It is found that the maximum temperature peak values (Figure 8) are registered by n-dodecane (Lapointe et al. [38]) and Fifth-Surr. These two peaks are responsible for the important exergy power and, therefore, better exergy efficiency. Figure 13 shows the exergy efficiency for various surrogates. It is worth noticing that 47.4% is the exergy efficiency obtained by the fifth reaction mechanism. The high temperature for the fifth reaction mechanism, n-dodecane (H.J Curran) and n-heptane, as shown in Figure 8, is responsible for the high exergy efficiency.

## 5. Conclusions

The present work examines the process of spraying and turbulent combustion in an internal diesel engine. The turbulent flow is described by the RNG k−ε turbulence model, and the spray atomization is governed by the Kelvin-Helmholtz/Rayleigh-Taylor Breakup model. The combustion processes of different fuel reaction mechanisms are numerically investigated and compared against experimental results. An analysis of exergy efficiency for all reaction mechanisms is carried out. Multi-component surrogate fuel is better used to emulate the physicochemical aspects of a commercial diesel fuel compared to the one-component reaction mechanism. Here, the chemistry models the transported species and chemical reactions, while the physics tracks the spray (e.g., injection, vaporization, droplet breakup). Multi-component surrogate fuel improves the prediction of fuel-oxidizer mixture preparation and better models the chemical reaction source term of the solved species. Thus, it pursues a more accurate reaction during the combustion in an IC engine.

These reaction mechanisms (listed below) are generated based on a validated detailed RM by the Ansys Model Fuel Library, i.e., Diesel_PAH_NOx_MFL2019, which involves 5384 species and 68,885 reactions:

(1) Predicted-1 (30% of aromatics and 70% n-alkanes): this RM has 1883 species and 8254 reactions;
(2) Predicted-2 (aromatics, cycloalkanes, iso-alkanes, and n-alkanes): this RM has 1883 species and 8254 reactions;
(3) Predicted-3 (aromatics, iso-alkanes, and n-alkanes): involves 1883 species and 8254 reactions;
(4) Predicted-4 (aromatics, 1 ether, 1 iso-alkane, and 2 composition n-alkanes): this RM has 445 species and 3562 reactions;
(5) Predicted-5 (1 component of aromatics, 1 cycloalkane, and 2 composition n-alkanes): this RM has 5384 species and 68,885 reactions.

These mechanisms are compared against n-dodecane (65 species with 363 reactions), n-heptane (770 reactions–159 species), and a diesel fuel surrogate (163 species with 887 reactions) from the literature.

The validation with experimental results includes pressure, HRR, and vapor penetration. The following points are registered:

- The fourth and fifth predicted surrogates show a better agreement with the experiment. They also produce lower pollutant concentrations than the other RMs.
- Cetane number (CN) plays a crucial role in combustion behavior. Lower cetane numbers (Predicted-1, -2, and -3) lead to an important ignition delay time, thus lowering the heat release rate (HRR). A higher CN results in a shorter ignition delay so that the HRR is increased. In opposition to pressure and heat release rate, a smaller variation of temperature is observed for all reaction mechanisms
- Vapor penetration length is not influenced by the surrogate fuel’s physical properties. This result is consistent with the experimental data.
- The highest exergy efficiency, 47%, is obtained by the fifth surrogate. This reaction mechanism produces an important pressure increase. The exergy variation of the different reaction mechanisms mimics well the behaviors of the pressure variations.

The computational time remains a challenge. The simulation using the predicted surrogate 5, involving 5384 species and 68,885 reactions, took two weeks and four days. The simulations were conducted on an Intel Xeon CPU E5-2650 v3 2.30 GHz with 16 CPUs.

## Figures and Tables

**Table 1:** Measured properties of baseline diesel fuel and predicted properties of the baseline surrogate fuel CN50_TSI31.

Fuel Property	Units	Baselines Diesel Fuel (Measured)	Baselines Diesel Fuel Surr. (Predicted-1)	Baselines Diesel Fuel Surr. (Predicted-2)	Baselines Diesel Fuel Surr. (Predicted-3)	Baselines Diesel Fuel Surr. (Predicted-4)	Baselines Diesel Fuel Surr. (Predicted-5)
**Cetane Number**		50.9	43.69	50.42	61.36	47.54	62.92
**Smoker Point**	mm	19.0					
**Threshold Soot Index**		31.0		31.1566	31.0956		27.0859
**Lower Heating Value**	MJ/kg	43.004	43.46	44.09	44.22	44.62	43.46
**Density at 15 °C**	g/mL	0.849	0.7565	0.8061	0.8033	0.7765	0.7819
**Kinetic Viscosity at 40 °C**	cSt	3.06		2.9263	2.9460	2.9819	1.2263
**Kinetic Viscosity at 40 °C**	cSt	0.99					
**Surface Tension**	N/m	0.0312					
**Lubricity—Wear Scar Diameter**	μm	489					
**T_10_**	°C	226.8		193.9	144.9	219.5	174.6
**T_90_**	°C	311.7		276.4	279	274.2	210.3
**Alkane Hydrocarbons**	%*v*/*v*	76.0					
**Alkene Hydrocarbons**	%*v*/*v*	7.5					
**Aromatic Hydrocarbons**	%*v*/*v*	16.5					
**Total Aromatics**	%m/m	16.4					
**Mono-Cyclic Aromatics**	%m/m	16.2					
**Polycyclic Aromatics**	%m/m	0.2					
**Carbon Content**	%m/m	86.38					
**Hydrogen Content**	%m/m	13.42					
**Sulfur Content**	ppm	9.4					
**H/C Molar Ratio**	molR	1.85	1.8344	1.9287	1.9252	2.1239	1.8764
**Stoichiometric A/F Ratio**		14.58					

**Table 2:** Specification parameters of the Sandia optical engine.

Item	Description
Engine base type	Cummins N-14, DI Diesel
Number of cylinders	1
Swirl ratio	0.5
Bore × Stroke	13.97 cm × 15.24 cm
Bowl width	9.78 cm
Bowl depth	1.55 cm
Geometric compression ratio	11.2:1
Simulated compression ratio†	16:1
Number of holes	8, equally spaced
Nozzle orifice diameter	0.196 mm

**Table 3:** Combustion strategies for the Sandia optical engine.

Item	Early-Inj. Low-T
Engine Speed (rpm)	1200
IMEP (bar)	3.9
Intake temperature (C)	90
Intake pressure (kPa)	214
SOI (CAD ATDC)	−22
Injection quantity (mg)	56
DOI	7
Conc. (Vol. %)	12.7

**Table 4:** Power and LHV of various surrogates and RMs.

Reaction Mechanism	n-Heptane (H.J. Curran [39])	N-Dodecane (Lapointe et al. [38])	Surr- (Pei et al. [1])	First-Surr.	Second-Surr.	Third-Surr.	Fourth-Surr.	Fifth-Surr.
Power [kW]	8.014	9.522	9.04366	5.502	6.632	7.778	9.1	10.34
Fuel LHV [kJ/g]	43.443	44.23	44.23	43.65	43.782	43.782	43.88	44.751

## Data Availability

Not applicable.

## Nomenclature

RM	Reaction Mechanism
RNG	Re-Normalization Group
HRR	Heat Release Rate
DI	Direct Injection
EVO	Exhaust Valve Opening
IVC	Intake Valve Closing
KH-RT	Kelvin-Helmholtz-Rayleigh Taylor
ICE	Internal Combustion Engine
DOI	Duration Of Injection
CI	Compression Ignition
CO_2_	Carbon Dioxide
CO	Carbon Monoxide
NO_x_	Nitric Oxides
LHV	Lower Heating Value
CA	Crank Angle
TDC	Top Dead Center
ATDC	After Top Dead Center
BTDC	Before Top Dead center
CN	Cetane Number
TSI	Threshold Soot Index
LTC	Low Temperature Combustion
UBHC	Unburned Hydrocarbon
RANS	Reynolds Averaged Navier-Stokes
SBO	Surrogate Blend Optimizer
CFD	Computational Fluid Dynamics
SOI	Start Of Injection
